# Transcultural Adaptation of the Oldenburg Burnout Inventory (OLBI) for Brazil and Portugal

**DOI:** 10.3389/fpsyg.2019.00338

**Published:** 2019-03-12

**Authors:** Jorge Sinval, Cristina Queirós, Sonia Pasian, João Marôco

**Affiliations:** ^1^William James Center for Research, ISPA – Instituto Universitário, Lisbon, Portugal; ^2^Faculty of Psychology and Education Sciences, University of Porto, Porto, Portugal; ^3^Faculty of Philosophy, Sciences and Languages of Ribeirão Preto, University of São Paulo, São Paulo, Brazil; ^4^Business Research Unit (BRU-IUL), Instituto Universitário de Lisboa (ISCTE-IUL), Lisbon, Portugal

**Keywords:** Oldenburg Burnout Inventory (OLBI), burnout, measurement invariance, Brazil, Portugal, multi-occupational, validity evidence

## Abstract

During the last few years, burnout has gained more and more attention for its strong connection with job performance, absenteeism, and presenteeism. It is a psychological phenomenon that depends on occupation, also presenting differences between sexes. However, to properly compare the burnout levels of different groups, a psychometric instrument with adequate validity evidence should be selected (i.e., with measurement invariance). This paper aims to describe the psychometric properties of the Oldenburg Burnout Inventory (OLBI) version adapted for workers from Brazil and Portugal, and to compare burnout across countries and sexes. OLBI's validity evidence based on the internal structure (dimensionality, reliability, and measurement invariance), and validity evidence based on relationships with other variables (work engagement) are described. Additionally, it aims presents a revision of different OLBI's versions—since this is the first version of the instrument developed simultaneously for both countries—it is an important instrument for understanding burnout between sexes in organizations. Data were used from 1,172 employees across two independent samples, one from Portugal and the other from Brazil, 65 percent being female. Regarding the OLBI internal structure, a reduced version (15 items) was obtained. The high correlation between disengagement and exhaustion, suggested the existence of a second-order latent factor, burnout, which presented measurement invariance for country and sex. Confirmatory factor analysis of the Portuguese OLBI version presented good goodness-of-fit indices and good internal consistency values. No statistically significant differences were found in burnout between sexes or countries. OLBI also showed psychometric properties that make it a promising and freely available instrument to measure and compare burnout levels of Portuguese and Brazilian employees.

## Introduction

Work organizations and labor relations all over the world are undergoing significant changes, with an impact on workers' lives and health, since the demands of modern working life are increasing pressure to levels never seen before (International Labour Office, 2016). Thus, the workforce must deal with a new landscape where psychosocial risks at work must be addressed (European Agency for Safety and Health at Work, 2018). Stress is a risk which, at extreme levels, can lead to burnout. Burnout has become a global concern, and work-related stress is a big challenge to organizations' performance and to their workers' health. Burnout levels vary depending on country, occupation, and individual characteristics, among which sex, is one of the most important factors (Purvanova and Muros, 2010). Burnout can affect any worker, with consequences not only in terms of health, safety, and well-being, but also for productivity, quality of service, and cost-effectiveness to the organization (Poghosyan et al., 2010; Carod-Artal and Vázquez-Cabrera, 2013). It is a severe reaction to occupational stress, having in its symptomatology changes to physical and psychological health and behavioral-motivational aspects, expressed through a reduction in job satisfaction or even a change of profession (Marques-Pinto et al., 2003). It is a syndrome (psychological in nature) that may occur when workers chronically face a stressful working environment and feel low resources to face high job demands (Maslach et al., 2001; Bakker and Demerouti, 2007; Maslach, 2015). The definition of Burnout has been expanded from a concept associated with human services professions to a concept related to all kinds of professions that can be affected (Lindblom et al., 2006).

From a historical perspective, burnout was initially considered as a psychological phenomenon in the USA, beginning with studies by the psychologist Freudenberger (1974) and the psychologist Maslach (1976). Despite some criticism (Bianchi et al., 2017) and the existence of several other related constructs, such as karōshi (
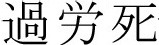
), meaning “death by overwork” (International Labour Organization, 2013) and karōjisatsu (
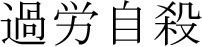
), meaning “suicide from overwork” (Amagasa et al., 2005), burnout became a popular topic in occupational health (Marques-Pinto et al., 2008; Schaufeli, 2017). There is some discussion about burnout (Bianchi, 2015; Bianchi et al., 2017; Epstein and Privitera, 2017; Mion et al., 2018) in terms of its dimensionality. The names attributed to the constructs can vary (Simbula and Guglielmi, 2010; Larsen et al., 2017). The most commonly suggested structure is a tri-factor one (Maslach et al., 2001, 2016), comprising emotional exhaustion (or simply exhaustion), depersonalization (also known as cynicism or disengagement), and reduced sense of personal accomplishment (or professional efficacy) (Halbesleben et al., 2004). It is expected that if a worker has high levels of the first two dimensions, there should be low levels of the third dimension since it is measured in the opposite direction to the other two. Carod-Artal and Vázquez-Cabrera (2013) state that emotional exhaustion is the most important dimension of burnout syndrome—being referred to as a state of having feelings of being emotionally overextended and depleted of one's emotional resources—representing the individual stress component (Bresó et al., 2007). Depersonalization refers to cynical or excessively detached responses to others in the work context; this is the interpersonal component of burnout (Maslach, 1998). Finally, diminished personal accomplishment refers to the decreased sense of competence and of productivity, representing the component of self-evaluation (Maslach, 1998).

There is also a two-dimensional approach to burnout (Demerouti et al., 2000). Based on empirical evidence, some authors consider that disengagement and exhaustion are the core dimensions of burnout, while reduced personal accomplishment plays a less important role (Maslach et al., 2001; Shirom, 2002). In fact, it has been shown that the relation of reduced personal accomplishment to burnout outcomes and antecedents is weaker than the other two dimensions (Lee and Ashforth, 1996). Moreover, while emotional exhaustion leads to disengagement, reduced personal accomplishment develops individually in relation to the other two dimensions (Leiter, 1993). Cordes and Dougherty (1993) suggest that it is an individual difference similar to self-efficacy.

Currently, burnout is becoming increasingly prominent in the literature (Leiter and Maslach, 2017); it has been associated with multiple occupational groups, beyond human services (Maslach and Leiter, 2017). In fact, burnout's prevalence has increased in some occupations, such as physicians in the USA (Shanafelt et al., 2015); it shows a high prevalence in various occupations, including radiology residents (Guenette and Smith, 2017), midwives in Australia (Creedy et al., 2017), nurses in various countries (Gómez-Urquiza et al., 2017). One reason why burnout is so common is due to the high levels of stress and emotional demands present in the job (Demerouti et al., 2001). Job stress can increase absenteeism, affect family roles, productivity, and mental and physical health, and decrease job satisfaction, which, in turn, can lead to reduced personal accomplishment, depersonalization, and emotional exhaustion (Carod-Artal and Vázquez-Cabrera, 2013).

### Burnout Measurement With the Oldenburg Burnout Inventory

Despite the existence of several instruments to measure burnout, the Maslach Burnout Inventory (MBI; Maslach et al., 2016) is the most used (Ahola et al., 2017) and is commercially available. However, there are other options, some of them free, such as the Copenhagen Burnout Inventory (Kristensen et al., 2005); the Burnout Measure (Pines and Aronson, 1988; Malach-Pines, 2005); the Educator Burnout Inventory (Wang et al., 2003), the Oldenburg Burnout Inventory (OLBI; Bakker et al., 2004); the Shirom-Melamed Burnout Measure (Shirom and Melamed, 2006); the Bergen Burnout Indicator (Salmela-Aro et al., 2011); the Karolinska Exhaustion Scale (Saboonchi et al., 2013); and the Spanish Burnout Inventory (Gil-Monte et al., 2017).

OLBI seems to be the most prominent alternative to MBI (Demerouti et al., 2000). It was originally developed by Demerouti and Nachreiner (1998), who suggested two burnout dimensions, disengagement and exhaustion, applicable to professionals outside human services occupations. OLBI's versions vary across occupational groups and countries (see Table 1). In some countries—Brazil and Portugal (Campos et al., 2012), Sweden (Dahlin et al., 2007; Rudman et al., 2014), Slovenia (Kogoj et al., 2014), South Africa (Mokgele and Rothmann, 2014), Germany and Greece (Reis et al., 2015), and Malaysia (Mahadi et al., 2018)—OLBI has a version for students. OLBI does not contain any factor correspondent to what the MBI calls “professional efficacy”; this dimension received criticism in some studies (Bresó et al., 2007; Marôco et al., 2014), and, in the opinion of various authors, it is not a core burnout dimension (Bakker et al., 2004; Demerouti and Bakker, 2008) but can be interpreted as a possible burnout consequence (Koeske and Koeske, 1989) related to personality characteristics (Cordes and Dougherty, 1993).

**Table 1:** OLBI's versions: validity evidence based on the internal structure.

**Country (Authors)**	**Occupational group**	***N***	**Dimensionality**		**Reliability: internal consistency**			**Measurement invariance**	**χ^2^/*df***	***TLI/NNFI***	***GFI***	***CFI***	***RMSEA***	***SRMR***
			**Items (factors)**	**Analysis**	**Total**	**Disengagement**	**Exhaustion**							
Brazil Schuster and Dias, 2018	Multi-occupational	273	16 (two)	CFA	–	*CR* = 0.93	*CR* = 0.89	–	2.59	0.91	0.90	0.93	0.07	0.06
			13 (two)		–	*CR* = 0.88	*CR* = 0.92	–	2.41	0.93	0.92	0.94	0.07	0.05
Cameroon Mbanga et al., 2018	Nurses	143	16 (two)	–	–	–	–	–	–	–	–	–	–	–
England Delgadillo et al., 2018	Psychological well-being practitioners	13	16 (two)	–	–	–	–	–	–	–	–	–	–	–
	Mental health nurse practitioners	15		–	–	–	–	–	–	–	–	–	–	–
	Cognitive behavioral therapists	21		–	–	–	–	–	–	–	–	–	–	–
	(Total)	(49)		–	–	α = 0.87	α = 0.84	–	–	–	–	–	–	–
Iraq Al-Asadi et al., 2018	Primary school teachers	706	16 (two)	–	–	–	–	–	–	–	–	–	–	–
Ireland Chernoff et al., 2018	Administrators	8	16 (two)	–	–	–	–	–	–	–	–	–	–	–
	Care assistants	3		–	–	–	–	–	–	–	–	–	–	–
	Nurses	50		–	–	–	–	–	–	–	–	–	–	–
	Physicians	23		–	–	–	–	–	–	–	–	–	–	–
	Porters	3		–	–	–	–	–	–	–	–	–	–	–
	Radiographers	10		–	–	–	–	–	–	–	–	–	–	–
	(Total)	(97)		–	–	–	–	–	–	–	–	–	–	–
Italy Estévez-Mujica and Quintane, 2018	Research and development	57	13 (two)	EFA	–	α = 0.86	α = 0.85	–	–	–	–	–	–	–
Malaysia Mahadi et al., 2018*	Medical students	452	16 (one)	CFA	–	–	–	–	7.606	0.577	0.768	0.633	0.121	–
			16 (two)		–	–	–	–	7.551	0.580	0.768	0.640	0.121	–
			9 (two)		α = 0.80	α = 0.74 *CR* = 0.73	α = 0.70 *CR* = 0.71	–	3.585	0.905	0.958	0.934	0.076	–
England Westwood et al., 2017	Psychotherapists	210	16 (two)	–	–	α = 0.83	α = 0.86	–	–	–	–	–	–	–
India Ananthram et al., 2017	Call center representatives	250	16 (two)	–	–	α = 0.84	α = 0.85	–	–	–	–	–	–	–
Kosovo Turtulla, 2017	Teachers	531	16 (two)	–	–	α = 0.73	α = 0.71	–	–	–	–	–	–	–
Malaysia Rosnah et al., 2017	Multi-occupational	492	8 (one)^E^	CFA	–	–	α = 0.50	–	3.21	–	0.98	0.92	0.066	–
Russia Smirnova, 2017	Multi-occupational	392	16 (one)	CFA	–	–	–	–	10.97	0.550	0.746	0.610	0.160	–
			16 (two)		–	α = 0.84	α = 0.65	–	9.60	0.612	0.804	0.709	0.148	–
			15 (two)		–	α = 0.84	α = 0.68	–	9.85	0.631	0.803	0.702	0.150	–
			7 (one)		–	–	–	–	8.72	0.802	0.911	0.868	0.141	–
			7 (two)		–	α = 0.65	α = 0.72	–	9.34	0.786	0.911	0.868	0.146	–
Saudi Arabia Al-shuhail et al., 2017	Physicians	140	16 (two)	–	–	–	–	–	–	–	–	–	–	–
Serbia Petrović et al., 2017	Multi-occupational	860	16 (two)	–	α = 0.81	–	–	–	–	–	–	–	–	–
Singapore Suyi et al., 2017	Health	37	16 (two)	–	–	α = 0.79–88	α = 0.63–89	–	–	–	–	–	–	–
Taiwan Ko, 2017	Hotel frontline employees	521	16 (two)	–	–	α = 0.75	α = 0.78	–	–	–	–	–	–	–
UK Halliday et al., 2017	Consultant	123	16 (two)	–	–	–	–	–	–	–	–	–	–	–
	General practitioner	93		–	–	–	–	–	–	–	–	–	–	–
	Higher specialist trainee	139		–	–	–	–	–	–	–	–	–	–	–
	Junior specialist trainee	153		–	–	–	–	–	–	–	–	–	–	–
	Foundation doctor	40		–	–	–	–	–	–	–	–	–	–	–
	(Total)	(548)		–	–	–	–	–	–	–	–	–	–	–
USA Olinske and Hellman, 2017	Executive directors	140	16 (two)	–	–	α = 0.78	α = 0.88	–	–	–	–	–	–	–
USA Yanos et al., 2017	Clinicians	472	16 (two)	–	α = 0.87	α = 0.75	α = 0.87	–	–	–	–	–	–	–
England Sales et al., 2016	General practitioners trainees	48	16 (two)	–	–	–	–	–	–	–	–	–	–	–
India Subburaj and Vijayadurai, 2016	Police constables	492	16 (one)	CFA	*CR* = 0.65	–	–	–	9.14	0.802	0.738	0.828	0.129	–
			16 (two)		–	*CR* = 0.91	*CR* = 0.90	–	3.75	0.933	0.911	0.942	0.075	–
	Higher secondary teachers	385	16 (one)		*CR* = 0.62	–	–	–	8.65	0.760	0.697	0.792	0.141	–
			16 (two)		–	*CR* = 0.90	*CR* = 0.90	–	3.64	0.917	0.902	0.929	0.083	–
	(Total)	(877)	–		–	–	–	–	–	–	–	–	–	–
Norway Innstrand, 2016	Advertising	301	16 (two)	CFA	–	–	–	–	3.38	0.96	–	0.96	0.092	0.069
	Bus drivers	381			–	–	–	–	3.21	0.97	–	0.97	0.083	0.051
	Church ministers	500			–	–	–	–	3.58	0.96	–	0.97	0.075	0.055
	IT	358			–	–	–	–	4.10	0.95	–	0.96	0.097	0.074
	Lawyers	412			–	–	–	–	3.66	0.96	–	0.97	0.084	0.056
	Nurses	496			–	–	–	–	4.87	0.96	–	0.97	0.092	0.064
	Physicians	523			–	–	–	–	5.58	0.94	–	0.95	0.098	0.072
	Teachers	504			–	–	–	–	4.86	0.96	–	0.96	0.091	0.067
	(Total)	(3,475)		MGCFA	–	α = 0.86–88	α = 0.87–88	Partial scalar invariance among occupational groups.	4.47	0.95	–	0.95	0.094	0.067
Pakistan Khan et al., 2016	Female academicians	299	16 (two)	–	–	–	–	–	–	–	–	–	–	–
Pakistan Khan and Yusoff, 2016	Academic staff	450	16 (four)	EFA	α = 0.83	–	–	–	–	–	–	–	–	–
			16 (one)	CFA		–	–	–	0.60	–	0.87	1.00	0.014	–
			16 (two)			–	–	–	2.62	–	0.99	0.98	0.004	–
Poland Baka and Basinska, 2016	Teachers	545	–	–	–	–	–	–	–	–	–	–	–	–
	Medical staff	491	–	–	–	–	–	–	–	–	–	–	–	–
	Police officers	768	–	–	–	–	–	–	–	–	–	–	–	–
	(Total)	(1,804)	16 (two)	EFA	–	α = 0.73	α = 0.69	–	–	–	–	–	–	–
USA Rogala et al., 2016	Health care	135	16 (two)	–	–	α = 0.86	α = 0.81–86	–	–	–	–	–	–	–
Zimbabwe Buitendach et al., 2016	Bus drivers	283	16 (two)	–	α = 0.76	α = 0.73	α = 0.72	–	–	–	–	–	–	–
Brazil Schuster et al., 2015	Multi-occupational	273	16 (two)	–	α = 0.90	α = 0.86	α = 0.83	–	–	–	–	–	–	–
			9 (two)	EFA	α = 0.88	α = 0.87	α = 0.76	–	–	–	–	–	–	–
Germany Reis et al., 2015	Nurses	385	15 (one)	CFA	–	–	–	–	5.28	0.82	–	0.84	0.11	0.07
			15 (two)		–	–	–	Partial metric invariance with German students	3.59	0.89	–	0.91	0.08	0.06
			16 (one)		–	–	–	–	5.26	0.80	–	0.82	0.11	0.07
			16 (two)		–	α = 0.81	α = 0.87	–	3.78	0.87	–	0.89	0.09	0.07
India Rajeswari and Sreelekha, 2015	Nurses	200	16 (two)	–	–	–	–	–	–	–	–	–	–	–
Poland Staszczyk and Tokarz, 2015	White-collar	210	16 (two)	–	α = 0.83	α = 0.78	α = 0.74	–	–	–	–	–	–	–
Slovenia Sedlar et al., 2015	Multi-occupational	1,436	8 (two)	CFA	–	–	–	–	7.51	0.988	–	0.992	0.067	–
			16 (one)		α = 0.83	–	–	–	39.94	0.753	–	0.786	0.165	–
			16 (two)^W^			–	–	–	8.38	0.953	–	0.960	0.072	–
			16 (two)			α = 0.71	α = 0.73	–	40.09	0.752	–	0.787	0.165	–
			16 (four)^PN^			–	–	–	8.06	0.955	–	0.963	0.070	–
USA Foster, 2015	Multi-occupational	579	8 (one)^D^	CFA	–	α = 0.82	–	Full-uniqueness measurement invariance between sex.	11.68	–	–	0.88	–	0.064
			8 (one)^E^	CFA	–	–	α = 0.84	Full-uniqueness measurement invariance between sex.	–	–	–	–	–	–
			16 (two)	–	–	–	–	–	–	–	–	–	–	–
USA Shupe et al., 2015	Librarians	282	16 (two)	–	α = 0.87	–	–	–	–	–	–	–	–	–
Sweden Lundkvist et al., 2014	Coaches	277	8 (two)^N^	CFA	–	–	–	–	3.25	0.940	–	0.959	0.090	–
			16 (two)		–	–	–	–	2.82	0.879	–	0.897	0.081	–
Sweden Rudman et al., 2014	Nurses	1,178	10 (two)^T^	–	–	α = 0.75	α = 0.78	–	–	–	–	–	–	–
		1,086	8 (two)^T^	–	–	α = 0.75	α = 0.71	–	–	–	–	–	–	–
		1,135	8 (two)^T^	–	–	α = 0.77	α = 0.71	–	–	–	–	–	–	–
		908	10 (two)^T^	–	–	α = 0.78	α = 0.80	–	–	–	–	–	–	–
		811	10 (two)^T^	–	–	α = 0.80	α = 0.81	–	–	–	–	–	–	–
Australia Scanlan and Still, 2013	Occupational therapists	34	16 (two)	–	–	α = 0.79	α = 0.84	–	–	–	–	–	–	–
Brazil Schuster et al., 2013	Multi-occupational	273	9 (two)	EFA	α = 0.88	α = 0.86	α = 0.76	–	–	–	–	–	–	–
			9 (two)	CFA	*CR* = 0.95	*CR* = 0.85	*CR* = 0.93	–	2.36	0.956	0.954	0.968	0.071	–
Poland Rzeszutek, 2013	Psychotherapists	200	16 (two)	–	α = 0.88	α = 0.82	α = 0.79	–	–	–	–	–	–	–
USA Ford et al., 2013	Information technology	91	16 (two)	–	–	α = 0.82	α = 0.79	–	–	–	–	–	–	–
South Africa Lekutle and Nel, 2012	Cement factory	187	5 (two)	EFA	–	α = 0.68	α = 0.69	–	–	–	–	–	–	–
Norway Innstrand et al., 2012	Multi-occupational	3,475	16 (two)	MGCFA	–	α = 0.86–88	α = 0.87–88	Longitudinal metric invariance.	–	–	–	–	–	–
China Qiao and Schaufeli, 2011	Nurses	717	16 (one)	CFA	–	–	–	–	11.62	0.73	0.75	0.76	0.11	–
			16 (two)^W^			–	–	–	5.58	0.88	0.90	0.90	0.08	–
			16 (two)			–	–	–	11.25	0.74	0.78	0.77	0.12	–
			16 (four)^PN^			–	–	–	4.42	0.91	0.92	0.93	0.07	–
Poland Baka, 2011	Teachers	292	16 (two)	–	α = 0.88	–	–	–	–	–	–	–	–	–
Sweden Peterson et al., 2011	Multi-occupational	3,719	16 (two)	CFA	–	α = 0.83	α = 0.83	–	27.88	0.93	–	0.94	0.08	0.06
			16 (one)		–	–	–	–	47.24	0.92	–	0.93	0.12	0.09
Poland Baka and Cieślak, 2010	Teachers	236	16 (two)	–	α = 0.87	–	–	–	–	–	–	–	–	–
South Africa Demerouti et al., 2010	Construction	528	16 (two)	–	–	α = 0.79	α = 0.74	–	–	–	–	–	–	–
South Africa Tilakdharee et al., 2010	Training and development	80	16 (two)	–	α = 0.928	α = 0.80	α = 0.82	–	–	–	–	–	–	–
Belgium Barbier et al., 2009	Public sector	955	16 (two)	–	–	α = 0.79	α = 0.82	–	–	–	–	–	–	–
Canada Chevrier, 2009	Catering	84	16 (five)	PCA	α = 0.80	–	–	–	–	–	–	–	–	–
			16 (two)	PCA		α = 0.69	α = 0.81	–	–	–	–	–	–	–
Netherlands Demerouti and Bakker, 2008	Health care	979	16 (two)	EFA	–	–	–	–	–	–	–	–	–	–
	White collar	644	16 (two)	EFA	–	–	–	–	–	–	–	–	–	–
	(Total)	1,623	16 (two)	CFA^T^	–	–	–	–	8.08	–	0.88	0.86	0.07	–
			16 (two)^W^	CFA^M^	–	–	–	–	12.51	–	0.76	0.76	0.08	–
			16 (two)	CFA^MTMM^	–	α = 0.85	α = 0.85	Metric invariance for burnout factors between occupations.	4.25	–	0.95	0.95	0.05	–
Sweden Peterson et al., 2008	Health care	3,719	16 (two)	–	–	α > 0.70	α > 0.70	–	–	–	–	–	–	–
Australia Timms et al., 2007	Teachers	298	16 (two)	–	–	α = 0.79	α = 0.81	–	–	–	–	–	–	–
South Africa Bosman et al., 2005	Government	297	16 (two)	–	–	α = 0.71	α = 0.66	–	–	–	–	–	–	–
United States of America Halbesleben and Demerouti, 2005	Multi-occupational	2,431	16 (one)	CFA	–	–	–	–	2.26	0.62	0.72	0.68	0.14	–
			16 (two)^W^		–	–	–	–	1.90	0.75	0.81	0.79	0.09	–
			16 (two)		–	α = 0.76–0.83	α = 0.74–0.79	–	1.09	0.96	0.97	0.95	0.03	–
	Fire department	168	16 (one)		–	–	–	–	2.66	0.75	0.71	0.78	0.14	–
			16 (two)^W^		–	–	–	–	2.93	0.71	0.68	0.74	0.16	–
			16 (two)		–	α = 0.83	α = 0.87	–	1.18	0.95	0.96	0.95	0.04	–
	(Total)	2,599	–	–	–	–	–	–	–	–	–	–	–	–
South Africa le Roux, 2004	Earthmoving	326	15 (two)	PCA	–	α = 0.82	α = 0.71	–	–	–	–	–	–	–
			16 (two)		–	–	–	–	–	–	–	–	–	–
Greece Demerouti et al., 2003	Multi-occupational	232	13 (one)	CFA	–	–	–	–	5.04	–	0.79	0.71	0.13	–
			13 (two)^W^			α = 0.83	α = 0.73	–	5.06	–	0.79	0.72	0.13	–
			13 (two)					–	3.39	–	0.87	0.83	0.10	–
			13 (two)^M^					–	1.90	–	0.94	0.95	0.062	–
Germany Demerouti et al., 2002	Human services	149	–	–	–	–	–	–	–	–	–	–	–	–
	Production employees	145	–	–	–	–	–	–	–	–	–	–	–	–
	(Total)	294	15 (one)	MGCFA	–	–	–	–	2.22	0.83	0.87	0.90	–	–
			15 (two)^U^			α = 0.84	α = 0.85	–	2.08	0.84	0.90	0.91	–	–
			15 (two)^W^					–	2.20	0.84	0.87	0.90	–	–
			15 (two)					–	1.26	0.91	0.93	0.99	–	–
German Demerouti et al., 2001	Human services	140	15 (one)	CFA	–	–	–	–	2.13	–	0.86	0.84	–	–
			15 (two) ^W^		–	–	–	–	2.10	–	0.87	0.85	–	–
			15 (two)		–	–	–	–	1.45	–	0.91	0.94	–	–
	Production	93	15 (one)		–	–	–	–	1.86	–	0.86	0.91	–	–
			15 (two) ^W^		–	–	–	–	1.73	–	0.87	0.92	–	–
			15 (two)		–	–	–	–	1.29	–	0.91	0.97	–	–
	Transport	119	15 (one)		–	–	–	–	1.52	–	0.86	0.86	–	–
			15 (two) ^W^		–	–	–	–	1.35	–	0.87	0.91	–	–
			15 (two)		–	–	–	–	1.14	–	0.90	0.96	–	–
	(Total)	352	15 (one)		–	–	–	–	3.73	–	0.87	0.88	–	–
			15 (two) ^W^		–	–	–	–	3.57	–	0.89	0.89	–	–
			15 (two)		–	α = 0.83	α = 0.82	–	1.50	–	0.96	0.98	–	–
			15 (one)	MGCFA	–	–	–	–	1.97	–	0.85	0.86	–	–
			15 (two) ^W^		–	–	–	–	1.90	–	0.86	0.87	–	–
			15 (two)		–	–	–	Metric invariance between occupations.	1.38	–	0.90	0.95	–	–
Germany Demerouti et al., 2000	Nurses	109	15 (two)	–	–	α = 0.92	α = 0.84	–	–	–	–	–	–	–
Germany Demerouti and Nachreiner, 1998	Service-Professionals	145	–	–	–	–	–	–	–	–	–	–	–	–
	Production	134	–	–	–	–	–	–	–	–	–	–	–	–
	Air traffic controllers	95	–	–	–	–	–	–	–	–	–	–	–	–
	(total)	374	25 (two)	EFA	–	α = 0.93	α = 0.82	–	–	–	–	–	–	–

* *Although a medical students sample was used, this version's items were adapted for workers; M, with modification indices applied; U, uncorrelated model; W, all positively phrased items of both burnout dimensions were specified to load on one factor and all negatively phrased items on a second factor; PN, two negatively worded scales (exhaustion items and disengagement items), and two positively worded scales (exhaustion items and disengagement items); D, disengagement subscale; E, exhaustion; N, only negatively worded items included, four each subscale; T, trait; M, method; MTMM, multitrait-multimethod*.

*The extracted results for the goodness-of-fit indices are presented with two or three decimal places depending of the original authors report. CFA, confirmatory factor analysis; EFA, exploratory factor analysis; MGCFA, multi-group confirmatory factor analysis; PCA, principal component analysis; GFI, goodness-of-fit index; RMSEA, root mean square error of approximation; NNFI, non-normed fit index; TLI, Tucker Lewis index; CFI, comparative fit index; SRMR, standardized root mean square residual*.

The exhaustion subscale of OLBI has eight items which relate to feelings of emptiness, work overload, the need to rest, and physical, cognitive, and emotional exhaustion (Demerouti et al., 2003). Differently from the exhaustion concept presented in the MBI, the OLBI approach to exhaustion covers cognitive, physical, and affective aspects of exhaustion, which may facilitate the use of the instrument with workers of different kinds of activity (Demerouti et al., 2003; Bakker et al., 2004). The disengagement subscale has also eight items which refer to distancing oneself from the work, together with negative and cynical behaviors and attitudes in relation to one's job (Demerouti and Bakker, 2008). The OLBI's concept of disengagement differs from MBI's depersonalization in terms of the amplitude of the distancing, since OLBI's concept is broader: it may refer to distancing oneself from work in general or, more specifically, to distancing oneself from the content and object, along with experiencing negative attitudes (Demerouti et al., 2003). Thus, disengagement offers a less restricted view of the lack of interest in work. It is important to note that González-Romá et al. (2006) suggested that two of the three work engagement dimensions measured by the Utrecht Work Engagement Scale (UWES), vigor and dedication (the third dimension is absorption), can be paired with emotional exhaustion and cynicism (burnout dimensions). One dimension, named “identification,” involved dedication and cynicism; the other, named “energy,” comprised vigor and exhaustion factors, this indicates that OLBI's negatively- and positively-worded items can be markers for work engagement and burnout (Halbesleben and Demerouti, 2005).

Table 1 summarizes the different OLBI versions used with different samples found through a search of Embase, Scopus, PubMed, Web of Science, and Google Scholar using the terms: “OLBI,” “Oldenburg Burnout Inventory,” “adaptation,” “version,” “validity,” and “psychometric properties.” The OLBI's total number of items changed since its original structure of 25 items (Demerouti and Nachreiner, 1998) to 15 (Demerouti et al., 2001); today's English language version has 16 items (Bakker et al., 2004; Halbesleben and Demerouti, 2005). It has positively and negatively worded items—an equal number of each kind in the two dimensions—something that is considered an advantage (Price, 1997) since it can diminish acquiescence bias despite diminishing the internal consistency of the instruments (Salazar, 2015). OLBI has been translated into many languages, although not always evaluated in its psychometric properties (Table 1). Some studies use OLBI without taking into consideration the recommended steps to adapt an instrument for a country or culture different from the one for which it was originally developed (van de Vijver, 2016). There is a certain lack of use of adequate guidelines when translating and adapting the instrument for a new sample (International Test Commission, 2018). As can be observed in Table 1, the majority of the new OLBI versions have not evaluated their psychometric properties with the appropriate technique (confirmatory factor analysis [CFA]; Brown, 2015). In fact, some of them have avoided both exploratory and confirmatory factor analysis. Usually, the original two-factor structure is the one with better goodness-of-fit indices—even when compared with the other two-factor structures (e.g., with positive items in one group and negatively-worded items in the other), and with one- or four-factor structures (Demerouti et al., 2001). The measurement invariance/equivalence of the instrument across different groups is essential to properly establish comparisons (Davidov et al., 2014). OLBI invariance has been addressed by Demerouti et al. (2001) in its 15-items version for three different professions, they observed metric invariance (same factor's loadings). Demerouti and Nachreiner (1998) stated that three different groups of professionals obtained a similar OLBI (25-items) structure after a principal component analysis for each of the groups. Others researchers have obtained measurement invariance between countries (Demerouti et al., 2003) and between workers and students (Reis et al., 2015). These findings suggest that burnout is not exclusive to human services professions (Demerouti and Nachreiner, 1998; Demerouti et al., 2001) since various studies have tested burnout levels using OLBI in other occupations (e.g., executive directors, white-collar employees, construction workers). Altogether, few studies tested the measurement invariance of the groups with which they established comparisons. Finally, regarding the reliability of the scores, the internal consistency estimates were acceptable to good in most of the studies, while almost all studies reported only the Cronbach's α (see Table 1).

This study aims to describe the psychometric properties of an OLBI version developed simultaneously for workers from Brazil and Portugal, its validity evidence based on the internal structure (dimensionality, measurement invariance, reliability), and the validity evidence based on the relationship with other variables (work engagement); and to compare burnout across sexes and countries. Additionally, the study seeks to present a revision of OLBI's different versions since this is the first version of the instrument developed simultaneously for Portugal and Brazil, adapting an important instrument for understanding burnout in relation to sexes in the organizations. It will be structured by presenting some considerations about burnout among sexes, followed by burnout measured by OLBI.

### Research Hypotheses

Following the recommendations of *The Standards for Educational and Psychological Testing* (American Educational Research Association, 2014), this paper aims to assess two types of validity evidence for the Portuguese version (PT-BR and PT-PT) of the OLBI (Bakker et al., 2004)—one related to the internal structure, the other based on the relations to other variables (work engagement). Since various studies have successfully confirmed the original two-factor structure of OLBI (Halbesleben and Demerouti, 2005; Peterson et al., 2011; Subburaj and Vijayadurai, 2016), it was hypothesized that the tested OLBI version would present a good fit confirming its original dimensionality of two factors (H1). Burnout has been hypothesized by some authors as a higher-order dimension (Taris et al., 1999; Shirom and Melamed, 2006; Marôco et al., 2008). Thus, a possible second-order latent factor, burnout, was tested for OLBI (H2). Through the review of the different versions (Table 1), the majority of the studies showed acceptable to very good reliability of the scores' evidence in terms of internal consistency (e.g., Demerouti and Bakker, 2008; Innstrand, 2016; Subburaj and Vijayadurai, 2016). Consequently, it was assumed that OLBI would present acceptable internal consistency reliability estimates (H3). Some studies found evidence of measurement invariance for OLBI between occupations (Demerouti et al., 2001; Demerouti and Bakker, 2008; Innstrand, 2016) and sex (Foster, 2015), but none investigated measurement invariance among workers of different countries. H4 hypothesized that OLBI will present evidence of measurement invariance between sexes and countries.

Research has found that burnout levels can vary among sexes, with females usually presenting slightly more exhaustion than males (Purvanova and Muros, 2010; Innstrand et al., 2011), females being more likely to experience burnout (Dimou et al., 2016). However, others suggest that research does not allow one to conclude any sex-specific risks (Seidler et al., 2014; Adriaenssens et al., 2015) considering that the burnout differences can be related with the levels of workload as well as care-load (Bekker et al., 2005; Langballe et al., 2011). Burnout can also vary among countries also (Poghosyan et al., 2010; Alexandrova-Karamanova et al., 2016; Jovanović et al., 2016). North American countries have a tendency to present higher exhaustion and disengagement levels than European countries—differences that can be related to cultural aspects (Maslach et al., 2001). However, regarding the Portugal-Brazil comparison, no differences were reported in a previous study (Dias et al., 2010). Occupations can play a substantial role in burnout levels (e.g., emotional challenges of working in the teaching or caregiving role) (Maslach et al., 2001). Altogether, it was hypothesized that burnout's latent means differ between sexes and countries (H5).

Work engagement is known to be a construct with strong correlations with burnout (Demerouti et al., 2010; Petrović et al., 2017), since both can be considered indicators of well-being (Bakker et al., 2014). Thus, the divergent validity evidence based on the relation to other variables, work engagement, was assessed (H6).

## Methods

### Sample

A total sample composed of 1,172 participants was collected by combining two independent samples: one sample of Brazilian workers in various occupations (*n* = 604), and one of Portuguese workers in various occupations (*n* = 568). Both samples completed the OLBI and the Utrecht Work Engagement Scale (Schaufeli and Bakker, 2003). Participation was anonymous and voluntary. The average age of the total sample was 35.47 years (*SD* = 9.95), with 65% being female. Workers' occupations were according to the International Standard Classification of Occupations ISCO-08 (International Labour Office, 2012)—mainly professionals or administrative support—and 73% of the sample were, at least, college graduates (Table 2). Regarding children, 59% had none; 45% reported being married or cohabiting.

**Table 2:** Sociodemographics, occupational group, and academic level for each country, and total.

	**Brazil (*n* = 604)** **Multi-occupational**	**Portugal (*n* = 568)** **Multi-occupational**	**Total (*n* = 1,172)**
**SOCIODEMOGRAPHICS**			
Age: *M* (*SD*)	35.11 (10.13)	35.83 (9.76)	35.47 (9.95)
Sex: Female %	67.23%	62.84%	65.07%
Children: Yes%	38.97%	42.61%	40.77%
**OCCUPATIONAL GROUP**			
Armed Forces Occupations	1.55	4.44	2.97
Managers	15.53	8.87	12.27
Professionals	36.12	53.63	44.70
Technicians and Associate Professionals	8.74	12.90	10.78
Clerical Support Workers	27.38	9.48	18.60
Services and Sales Workers	6.21	6.05	6.13
Skilled Agricultural, Forestry and Fishery Workers	–	–	–
Craft and Related Trades Workers	2.14	2.22	2.18
Plant and Machine Operators and Assemblers	0.78	0.60	0.69
Elementary Occupations	1.55	1.81	1.68
**ACADEMIC LEVEL**			
PhD	5.12	5.64	5.38
Master	9.49	38.52	23.82
Post-graduation (not master neither PhD)	25.62	9.34	17.58
Graduation	34.16	29.57	31.89
Unfinished graduation	13.09	4.67	8.93
High school, vocational education or less	12.52	12.26	12.40

A non-probabilistic convenience sampling was used. The inclusion criteria were: (1) all participants were workers with a contract or formal ties with their employers, (2) had easy access to a PC, smartphone, or tablet to access the online platform where the instruments were deployed, and (3) were literate.

### Measures

The OLBI was used to assess burnout, through the development of a version transculturally adapted both for Brazil and Portugal (Table 3). The OLBI is a self-report five-point rating scale (1 = “Strongly disagree”; to 5 = “Strongly agree”) with eight questions within each of the two dimensions, disengagement and exhaustion (Demerouti et al., 2001). The disengagement factor refers to distancing from work in terms of both object and content, and to the development of cynical and negative attitudes and behaviors in relation to one's job (Bakker et al., 2004). Exhaustion refers to feelings of physical fatigue, the need to rest, and feelings of overtaxing and emptiness in relation to work (Demerouti and Bakker, 2008). To develop the Portuguese version (Table 3) the English version of the OLBI was used (Bakker et al., 2004) following *The ITC Guidelines for Translating and Adapting Tests* (International Test Commission, 2018), adapting the items to the Portuguese language according to the *Orthographic Agreement* signed by both Portugal and Brazil in 2009. The items were discussed with Portuguese and Brazilian psychologists and methodologists to create a version of the items that gathered the consensus of specialists regarding cultural, semantic, and idiomatic equivalence in the two countries. Finally, a small pilot test was done with 15 workers from each country; this did not suggest any modifications and the Portuguese adapted OLBI's 16 items were understood. The final single version (for both countries) had no other changes.

**Table 3:** OLBI original and Portuguese versions.

**Item**	**Original OLBI (Bakker et al.**, **2004****)**					**Portuguese (Brazil and Portugal) version of OLBI**				
	**Strongly disagree**	**Disagree**	**Neutral**	**Agree**	**Strongly agree**	**Discordo totalmente**	**Discordo**	**Nem concordo, nem discordo**	**Concordo**	**Concordo totalmente**
	**1**	**2**	**3**	**4**	**5**	**1**	**2**	**3**	**4**	**5**
	**DISENGAGEMENT**					**DISTANCIAMENTO**				
1^R^	I always find new and interesting aspects in my work					Encontro com frequência assuntos novos e interessantes no meu trabalho				
3	It happens more and more often that I talk about my work in a negative way					Cada vez mais falo de forma negativa do meu trabalho				
6	Lately, I tend to think less at work and do my job almost mechanically					Ultimamente tenho pensado menos no meu trabalho e faço as tarefas de forma quase mecânica				
7^R^	I find my work to be a positive challenge					Considero que o meu trabalho é um desafio positivo				
9	Over time, one can become disconnected from this type of work					Com o passar do tempo, sinto-me desligado do meu trabalho				
11	Sometimes I feel sickened by my work tasks					Às vezes, sinto-me farto das minhas tarefas no trabalho				
13^R^	This is only type of work that I can imagine myself doing					*Este é o único tipo de trabalho que me imagino a fazer				
15^R^	I feel more and more engaged in my work					Sinto-me cada vez mais empenhado no meu trabalho				
	**EXAUSTÃO**					**EXAUSTÃO**				
2	There are days when I feel tired before I arrive at work					Há dias em que me sinto cansado antes mesmo de chegar ao trabalho				
4	After work, I tend to need more time than in the past in order to relax and feel better					Depois do trabalho, preciso de mais tempo para relaxar e sentir-me melhor do que precisava antigamente				
5^R^	I can tolerate the pressure of my work very well					Consigo aguentar bem a pressão do meu trabalho				
8	During my work, I often feel emotionally drained					Durante o meu trabalho, muitas vezes sinto-me emocionalmente esgotado				
10^R^	After working, I have enough energy for my leisure activities					Depois do trabalho, tenho energia suficiente para minhas atividades de lazer				
12	After my work, I usually feel worn out and weary					Depois do trabalho sinto-me cansado e sem energia				
14^R^	Usually, I can manage the amount of my work well					De uma forma geral, consigo administrar bem a quantidade de trabalho que tenho				
16^R^	When I work, I usually feel energized					Quando trabalho, geralmente sinto-me com energia				

*R, reversed*;

* *Removed item for the proposed Portuguese (Brazil and Portugal) version*.

Work engagement refers to a positive motivational state and is composed of vigor, dedication, and absorption. This construct was measured with UWES-9 in its transculturally adapted version to both Brazil and Portugal (Sinval et al., 2018). It is a self-report instrument scored on a seven-point rating scale (0 = “Never”; 6 = “Always”), with three questions in each of its three dimensions. The UWES has shown good divergent validity evidence with the OLBI, since work engagement and burnout are moderately and negatively related (Goering et al., 2017). It was chosen not only for its good psychometric qualities for both countries, but also because it showed measurement invariance between both countries and it is a short instrument that allows for a robust work engagement measure with only a few items (Schaufeli and Bakker, 2003). It is a well-spread measure across many countries (Sinval et al., 2018) and is actually the most used instrument to measure work engagement. However, studies that investigated the relations between burnout and work engagement have mainly used the MBI for burnout and the UWES for work engagement (Schaufeli and de Witte, 2017). This study used OLBI together with UWES, trying to enrich the discussion about the two concepts, rather than just discussing instruments. The UWES dimensions are vigor, referring to the energy and resilience that one has in work; dedication, referring to being enthusiastic, inspired, and proud of one's work; and absorption, referring to being immersed in one's work without the perception of time passing (Schaufeli et al., 2002). It is expected that high levels of work engagement correspond to highly energized workers (Schaufeli and Bakker, 2010).

### Procedures

Data were gathered from 2015 to 2017, in both countries, in an effort to have a larger sample, since web surveys present low response rates (Massey and Tourangeau, 2013). Both samples completed the OLBI, a brief sociodemographic questionnaire, and the UWES-9. All the collected data were obtained online using *LimeSurvey* software (LimeSurvey GmbH, 2017) running on the website of two major universities in each country. Nearly 35 percent of the disseminated questionnaires were completed in both countries. Participants were both contacted individually and through companies which answered positively to the invitation to participate in the study. Before filling out the survey, participants were informed about the study, assuring them that the study was a research study and that the company would not access individual data and that companies simply helped the researchers disseminate the study. Informed consent was obtained online from all participants.

To allow comparative studies, the same procedures were used in both countries. The study was approved by the Ethics Committee of the University of Porto (on 03-18-2015), Portugal, and the University of São Paulo (on 01-09-2014; CAAE no. 33301214.2.0000.5407), Brazil, and followed the usual rules for online surveys, namely, no access of participants' companies to individual results and no direct contact between participants and researchers [A few used the email to clarify some details about access to individual data, but it is not possible to identify whether they participated in the study].

### Data Analysis

A confirmatory factor analysis (CFA) was conducted to verify if the original two-factor structure proposed by Bakker et al. (2004), presented an adequate fit to the study sample. Only complete data cases were considered. As goodness-of-fit indices, SRMR (Standardized Root Mean Square Residual), RMSEA (root mean square error of approximation), NFI (Normed Fit Index), CFI (Comparative Fit Index), and the TLI (Tucker Lewis Index) were used. The fit of the model was considered good for TLI, CFI and TLI values above 0.95; SRMR below 0.08; and RMSEA values below 0.08 (Hoyle, 1995; Boomsma, 2000; McDonald and Ho, 2002; Byrne, 2010). All statistical analyses were performed with *R* (R Core Team, 2018) and *RStudio* (RStudio Team, 2018). The descriptive statistics were obtained with the *skimr* package (McNamara et al., 2018), the standard error of the mean (SEM) was calculated with the *plotrix* (Lemon, 2006) package and the coefficient of variation (CV) was estimated with the package *sjstats* (Lüdecke, 2019). To assess multivariate normality, Mardia's multivariate kurtosis (Mardia, 1970) was used; it was calculated using the *psych* package (Revelle, 2018). The *lavaan* package (Rosseel, 2012) was selected to conduct the CFA analyses using the Weighted Least Squares Means and Variances (WLSMV) estimation method (Muthén, 1983).

To test the proposed structure for OLBI, the cross-validity evidence was assessed to give information about how well the new structure will fit an independent sample of the same population (Cudeck and Browne, 1983). To do so, the sample was randomly split into two sub-samples through the package *minDiff* (Papenberg, 2018). The workers' age was used as criteria variable for which it was desired to minimize differences between subsamples (Papenberg, 2018). The subsamples were generated using 1,000 repetitions in order to minimize the differences, since the most equal group assignment was selected. Having two independent subsamples with similar properties, one subsample can be used as calibration subsample, and another as validation subsample (Chin and Todd, 1995).

The convergent validity evidence was analyzed using the average variance extracted (AVE) which was estimated as described in Marôco (2014) and Fornell and Larcker (1981). The constructs' convergent validity evidence was assumed for values of AVE ≥ 0.5 (Hair et al., 2009).

The discriminant validity evidence was checked (Fornell and Larcker, 1981; Marôco, 2014) to verify whether the items that represent a dimension were strongly correlated with other dimensions (Marôco, 2014): for two factors, *x* and *y*, if AVE*x* and AVE*y* ≥ ρ^2^_*xy*_ (squared correlation between the factors *x* and *y*), there is discriminant validity evidence. The Heterotrait-monotrait (HTMT) criterion (Henseler et al., 2015) was also used. Values above 0.85 were considered indicative of satisfactory discriminant validity evidence (Kline, 2016). The HTMT ratios of correlations were calculated using the *semTools* package (Jorgensen et al., 2018).

The reliability of the scores was assessed with various estimates of internal consistency as recommended (Irwing and Hughes, 2018): α_*ordinal*_ (Zumbo et al., 2007), and ω_*ordinal*_ (Bollen, 1980; Raykov, 2001) using the *semTools* package (Jorgensen et al., 2018), higher values were indicative of better internal consistency results. Also, the McDonald's hierarchical omega (ω_*H*_; Zinbarg et al., 2005) was estimated; a higher value of ω_*H*_ indicates a stronger influence of the latent variable common to all factors, and that the observed scale scores generalize to scores for the common latent variable (Zinbarg et al., 2007). The omega hierarchical subscale (ω_*HS*_) was calculated for each specific factor, it reflects the reliability of each subscale after controlling for the variance due to the general factor (Reise et al., 2013). Both the ω_*H*_ and the ω_*HS*_ were used for calculating the internal consistency of the bi-factor model. There is some discussion about the use of α_*ordinal*_ (Revelle and Condon, 2018) as so we reported other estimates. The α_ordinal_ was calculated based on the polychoric correlations. However, the ω_*ordinal*_ and ω_*H*_ accounts for both item covariances and item thresholds (Green and Yang, 2009). The ω_*ordinal*_ and the ω_*H*_ are different in the denominator, the first assumes a congeneric factor model where measurement errors aren't correlated (Bollen, 1980), the second uses the observed covariance matrix instead of the model-implied covariance matrix (McDonald, 1999; Jorgensen et al., 2018). The CR was calculated by summing the z scores of the item scores. The second-order factor reliability was also calculated using the omega coefficient (Jorgensen et al., 2018). The proportion of observed variance explained by the second-order factor after controlling for the uniqueness of the first-order factor (ω_*partial L*1_); the proportion of the second-order factor explaining the variance of the first-order factor level (ω_*L*2_); and the proportion of the second-order factor explaining the total score (ω_*L*1_) were also calculated. The reliability estimates were calculated with the *semTools* package (Jorgensen et al., 2018).

The measurement invariance of the higher-order model was assessed using the *lavaan* package (Rosseel, 2012), the categorical items were considered into account through theta-parameterization (Millsap and Yun-Tein, 2004) to compare a group of seven different models based on the recommendations of Millsap and Yun-Tein (2004) and on the second-order models' invariance specificities (Chen et al., 2005): (a) configural invariance; (b) first-order factor loadings; (c) second-order structural loadings; (d) thresholds of measured variables; (e) intercepts of first-order factors; (f) disturbances of first-order factors; and (g) residual variances of observed variables. Mean scores for burnout latent variable were compared within the structural equation modeling framework; effect sizes (Cohen's *d*) were determined (Cohen, 1988). The raw means, SDs and score percentiles were calculated using the *doBy* package (Højsgaard and Halekoh, 2018).

## Results

The results related to psychometric properties of the OLBI in terms of internal structure are presented first, followed by the latent means comparisons, and finally by the validity evidence based on the relations to other variables.

### Validity Evidence Based on Internal Structure

#### Dimensionality

##### Items' distributional properties

To judge distributional properties and psychometric sensitivity on the Portuguese and Brazilian samples, summary measures, skewness (Sk), kurtosis (Ku), and a histogram for each of the 16 items were used (Table 4). No strong deviations from the normal distribution (Finney and DiStefano, 2013) were considered for absolute values of Ku smaller than seven (7) and Sk smaller than three (3), assuring that they wouldn't compromise CFA results (Marôco, 2014). Mardia's multivariate kurtosis for the 16 items of OLBI was 48.88; *p* < 0.001. All possible Likert-scale answer values were observed on all items; no outliers were deleted. These items follow an approximately normal distribution in the normative population under study, since their distributional properties are indicative of appropriate psychometric sensitivity.

**Table 4:**
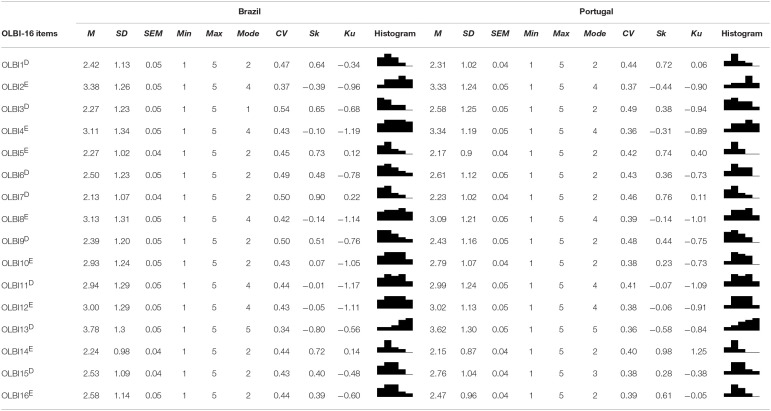
OLBI's items: descriptive statistics.

*D, disengagement items; E, exhaustion items*.

##### Factor-related validity evidence

**Figure 1:**
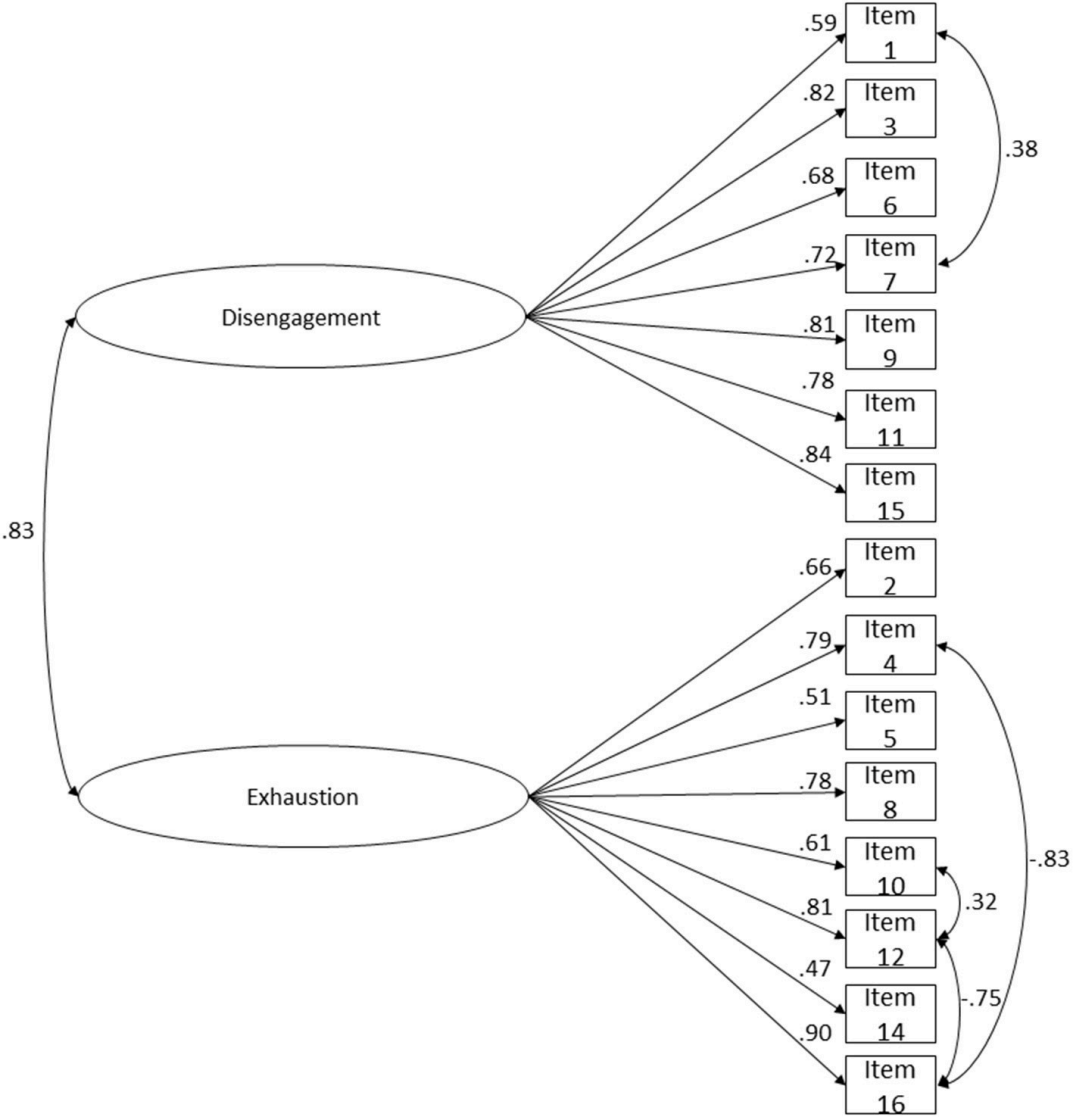
OLBI's two-factor reduced version (15-item) structure fit. A combined sample of Portuguese (*n* = 268) and Brazilian (*n* = 318) workers. Correlations between latent variables, residuals' correlations and factor loadings for each item are shown. $\chi_{\left(85\right)}^{2}$ = 514.098; *p* < 0.001; *n* = 586; *CFI* = 0.986; *CFI*_*scaled*_ = 0.937; *NFI* = 0.984; *TLI* = 0.983; *SRMR* = 0.064; *RMSEA* = 0.093; *P*(rmsea ≤ 0.05) < 0.001; 90% CI ]0.085; 0.101[.

To proceed with OLBI's transcultural adaptation to Brazil and Portugal, a cross-validity evidence approach was adopted. The sample was divided in two subsamples: calibration (*n* = 586) and validation subsamples (*n* = 586). The first was used to test which is OLBI's best solution in terms of fit to the data, and theoretical sense. The second subsample was used to assess cross-validity evidence of the proposed model. The two-factor OLBI fit to the data was mediocre ($\chi_{\left(103\right)}^{2}$ = 720.764; *p* < 0.001; *n* = 586; *CFI* = 0.980; *CFI*_*scaled*_ = 0.918; *NFI* = 0.977; *TLI* = 0.977; *SRMR* = 0.072; *RMSEA* = 0.101; *P*(rmsea ≤ 0.05) < 0.001; 90% CI ]0.094; 0.108[), since CFI, NFI, and TLI values were above 0.95 (good fit), SRMR values were bellow 0.08 (good fit), but RMSEA values were above 0.10 being indicative of poor fit (MacCallum et al., 1996). One item presented a very low loading (λ_item 13_ = 0.220): and thus, this item was deleted. Also, based on the analysis of the modification indices, four correlations between items' residuals of the same factor were added, since it seems reasonable that indicators from the same factor explain shared error variance (Kline, 2016). The reduced model of 15 items showed better goodness-of-fit indices (Figure 1; $\chi_{\left(85\right)}^{2}$ = 514.098; *p* < 0.001; *n* = 586; *CFI* = 0.986; *CFI*_*scaled*_ = 0.937; *NFI* = 0.984; *TLI* = 0.983; *SRMR* = 0.064; *RMSEA* = 0.093; *P*(rmsea ≤ 0.05) < 0.001; 90% CI ]0.085; 0.101[), which indicated an acceptable fit, all items presented loadings above or equal to 0.47 (*p* < 0.001). The Cheung and Rensvold (2002) criterion (Δ*CFI* ≤ 0.01) supported the preference for the reduced model (Δ*CFI*_*scaled*_ = −0.019). Thus, H1 was accepted.

##### Convergent validity evidence

To check if items contained within each factor are related to each other, the AVE was calculated for disengagement (*AVE* = 0.57), and for exhaustion (*AVE* = 0.50). These results suggest acceptable convergent validity evidence for the OLBI-15.

##### Discriminant validity evidence

The discriminant validity evidence between the two OLBI factors was unsatisfactory. These findings showed that the two factors are strongly related to each other, since *AVE*_*disengagement*_ = 0.57 and *AVE*_*exhaustion*_ = 0.50 were smaller than *r*^2^_*DE*_ = 0.69. Regarding the HTMT ratio of correlations (Henseler et al., 2015) the obtained value (0.80) is below the satisfactory threshold. These findings point to the fact that the two factors' correlation might be explained by a second-order latent factor, by a bi-factor model or by a unidimensional model.

##### Unidimensional model

A unidimensional model where the factor burnout loads on all 15 items was tested. The four residuals' correlations were maintained. This model assumes that the only latent factor that explains the manifest variables is *burnout*. As so, it assumes that the other two latent variables (i.e., *disengagement* and *exhaustion*) aren't meaningful by themselves since the discriminant validity evidence wasn't satisfactory. The content explained by them is similar, the unidimensional model tests if it is plausible to specify a single latent variable.

**Figure 2:**
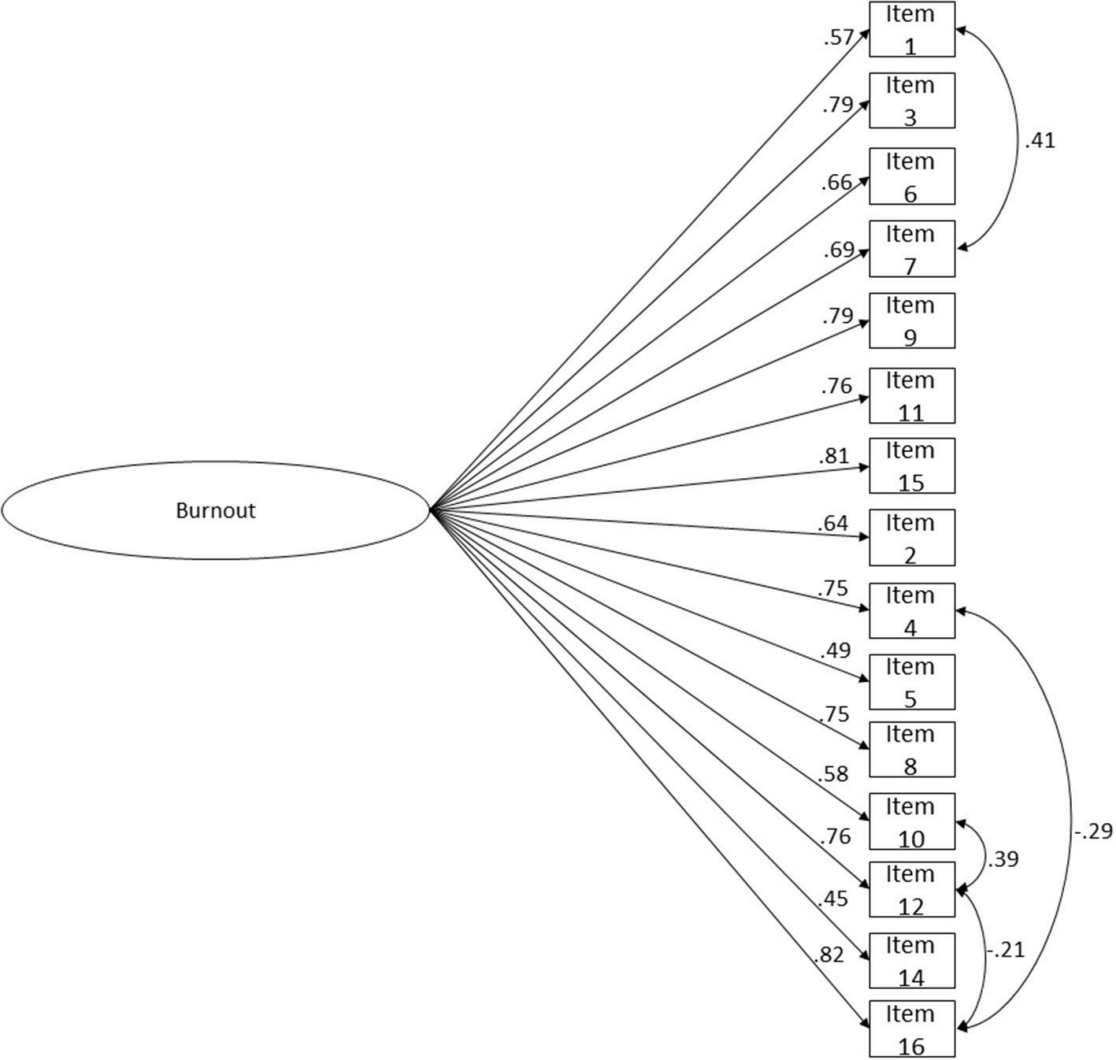
OLBI's unidimensional reduced version (15 items) structure fit. A combined sample of Portuguese (*n* = 268) and Brazilian (*n* = 318) workers. Residuals' correlations and factor loadings for each item are shown. $\chi_{\left(86\right)}^{2}$ = 737.139; *p* < 0.001; *n* = 586; *CFI* = 0.979; *CFI*_*scaled*_ = 0.913; *NFI* = 0.977; *TLI* = 0.975; *SRMR* = 0.077; *RMSEA* = 0.114; *P*(rmsea ≤ 0.05) < 0.001; 90% CI ]0.106; 0.121[.

The OLBI's unidimensional model presented an mediocre fit (Figure 2; $\chi_{\left(86\right)}^{2}$ = 737.139; *p* < 0.001; *n* = 586; *CFI* = 0.979; *CFI*_*scaled*_ = 0.913; *NFI* = 0.977; *TLI* = 0.975; *SRMR* = 0.077; *RMSEA* = 0.114; *P*(rmsea ≤ 0.05) < 0.001; 90% CI ]0.106; 0.121[). Based on the Cheung and Rensvold (2002) criteria (Δ*CFI* ≤ 0.01) the two-factor reduced model was found to have a statistically better fit to these data than the unidimensional model (Δ*CFI*_*scaled*_ = −0.024). All the factor loadings and residuals' correlations were statistically significant (*p* < 0.001). Item 14 had the lowest factor loading (λ_*item* 14_ = 0.450).

##### Bi-factor^1^ model

A bi-factor model (Holzinger and Swineford, 1937; Holzinger and Harman, 1938) is a *nested factor model* (Gustafsson and Balke, 1993) or *direct hierarchical model* (Gignac, 2008) that specifies a single *general* factor among each measured variable that accounts for commonality shared by the related domains; and multiple *specific* orthogonal factors each of which account for unique variance above and over the general factor (Rios and Wells, 2014; Mansolf and Reise, 2017; Chen and Zhang, 2018). The bi-factor model has advantages (Canivez, 2016; Chen and Zhang, 2018), but also some limitations (Mulaik and Quartetti, 1997; Reise et al., 2010; Murray and Johnson, 2013) in comparison with higher-order models (e.g., second-order models), as so, the choice between them should be carefully weighted.

**Figure 3:**
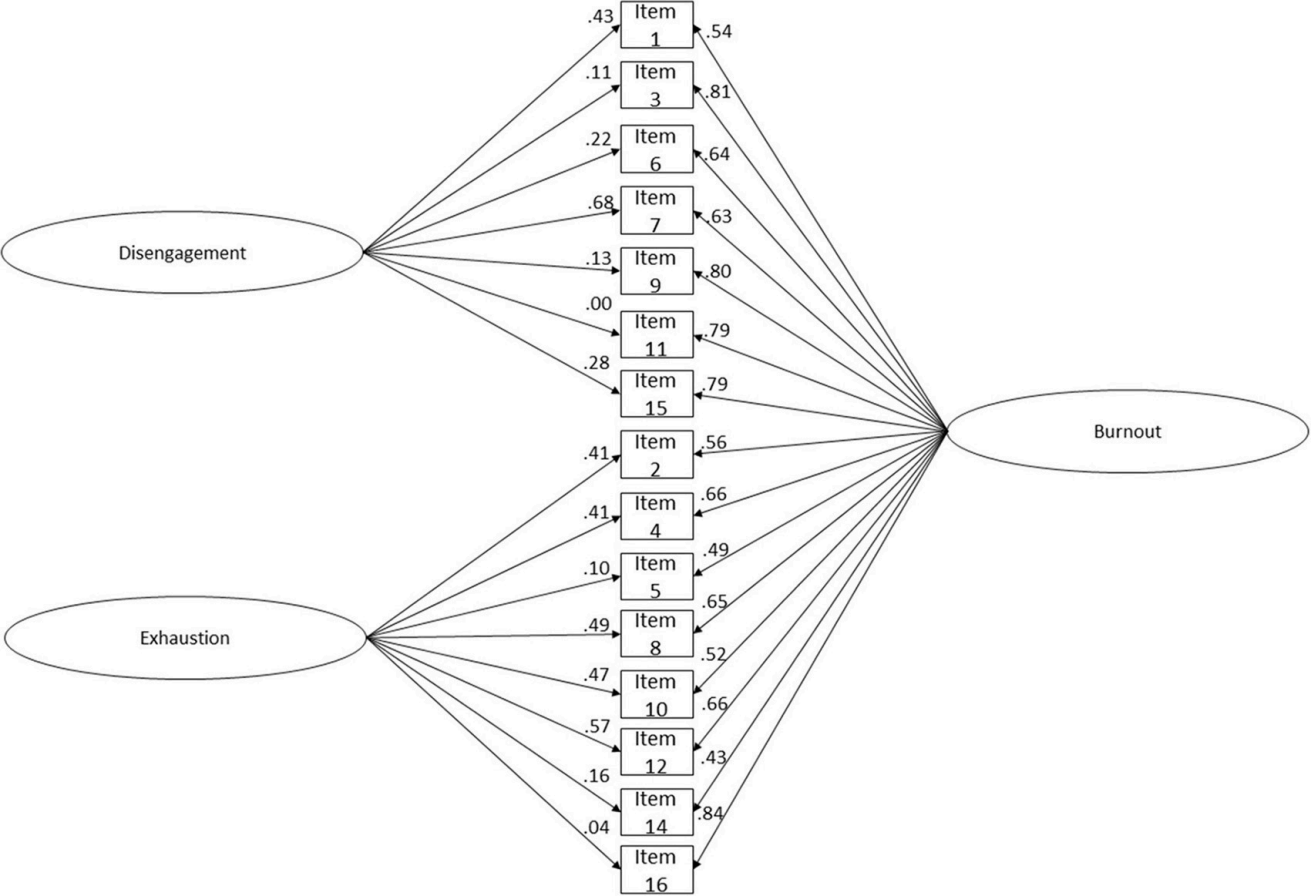
OLBI's bi-factor reduced version (15 items) structure fit. A combined sample of Portuguese (*n* = 268) and Brazilian (*n* = 318) workers. Latent loadings for each factor; and factor loadings for each item are shown. $\chi_{\left(75\right)}^{2}$ = 392.202; *p* < 0.001; *n* = 586; *CFI* = 0.990; *CFI*_*scaled*_ = 0.937; *NFI* = 0.987; *TLI* = 0.986; *SRMR* = 0.056; *RMSEA* = 0.085; *P*(rmsea ≤ 0.05) < 0.001; 90% CI ]0.077; 0.093[.

The OLBI's bi-factor model presented an acceptable fit (Figure 3; $\chi_{\left(75\right)}^{2}$ = 392.202; *p* < 0.001; *n* = 586; *CFI* = 0.990; *CFI*_*scaled*_ = 0.937; *NFI* = 0.987; *TLI* = 0.986; *SRMR* = 0.056; *RMSEA* = 0.085; *P*(rmsea ≤ 0.05) < 0.001; 90% CI ]0.077; 0.093[). The Δ*CFI* ≤ 0.010 criterion (Cheung and Rensvold, 2002) didn't find *meaningful* differences between the two-factor reduced model and the bi-factor model (Δ*CFI*_*scaled*_ = 0.000). All factor loading of the general factor (i.e., *burnout*) were statistically significant (*p* < 0.001), although the *specific* factors presented two non-significant loadings (α = 0.05), one on the *disengagement* subscale (λ_*item* 11_ = −0.001), and one on the *exhaustion* subscale (λ_*item* 16_ = 0.037).

##### Second-order model

A second-order latent factor may be admissible when two factors have high correlations between them, or/and when exists a higher order construct which might explain the lower order factors (Chen et al., 2005; Marôco, 2014). Since the two OLBI factors did not present satisfactory discriminant validity evidence between them, a second-order model was tested. The higher-order construct was named as *burnout*. Having as a start point the reduced model, and since there were not enough degrees of freedom to test the second-order latent model, the two structural weights between the second-order factor and the first-order factors were constrained to be equal.

**Figure 4:**
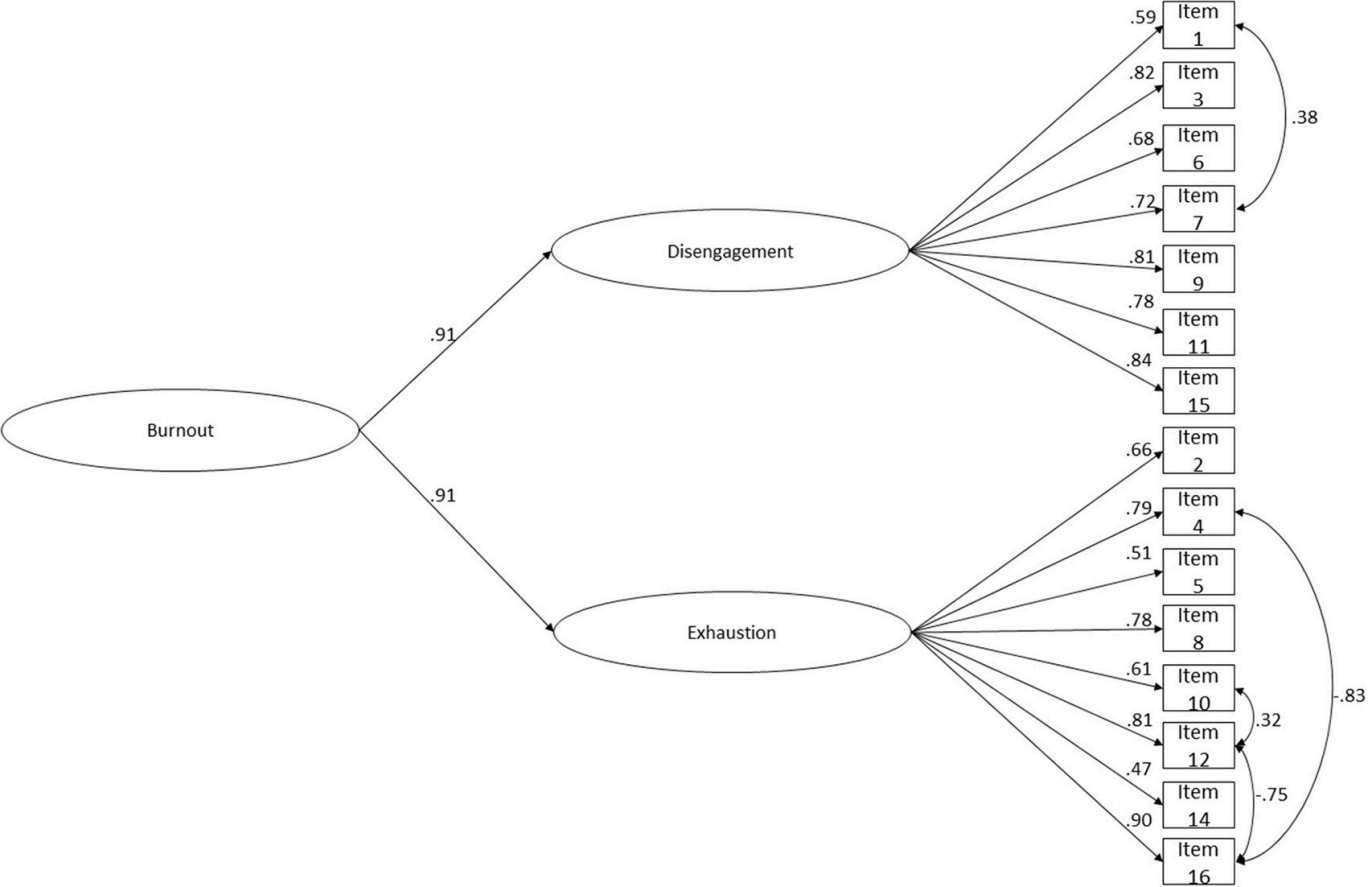
OLBI's second-order factor reduced version (15 items) structure fit. A combined sample of Portuguese (*n* = 268) and Brazilian (*n* = 318) workers. Latent loadings for each factor; residuals' correlations and factor loadings for each item are shown. $\chi_{\left(85\right)}^{2}$ = 514.098; *p* < 0.001; *n* = 586; *CFI* = 0.986; *CFI*_scaled_ = 0.937; *NFI* = 0.984; *TLI* = 0.983; *SRMR* = 0.064; *RMSEA* = 0.093; *P*(rmsea ≤ 0.05) < 0.001; 90% CI ]0.085; 0.101[.

The OLBI's second-order latent factor model presented an acceptable fit (Figure 4; $\chi_{\left(85\right)}^{2}$ = 514.098; *p* < 0.001; *n* = 586; *CFI* = 0.986; *CFI*_*scaled*_ = 0.937; *NFI* = 0.984; *TLI* = 0.983; *SRMR* = 0.064; *RMSEA* = 0.093; *P*(rmsea ≤ 0.05) < 0.001; 90% CI ]0.085; 0.101[). The RMSEA value was mediocre, however its confidence interval was precise and point estimates for RMSEA have been shown to depend on sample size and model misspecification and model degrees of freedom (MacCallum et al., 1996; Chen et al., 2008). Nevertheless, other goodness-of-fit indices were used in conjunction to assess models' adequacy. SRMR values were acceptable, which seem to be generally accurate across all conditions (Maydeu-Olivares et al., 2018). The constrained structural weights from burnout to disengagement and exhaustion were high (γ = 0.91; *p* < 0.001). These results suggest that burnout is a higher order construct reflected on disengagement and exhaustion. The findings show that hypothesis 2 can be confirmed, since the paths from the second-order latent to the first-order ones were statistically significant (*p* < 0.001) and had high values.

The differences between the second-order model and the two-factor reduced model (Δ*CFI*_*scaled*_ = 0.000) and the bi-factor model (Δ*CFI*_*scaled*_ = 0.000) weren't *meaningful* based on the ΔCFI criteria (Cheung and Rensvold, 2002). After having in consideration all tested models (Table 5) the second-order model was selected, since it hadn't a worst fit than the bi-factor and the two-factor reduced model (based on the used criterion). This choice emerged as solution for the lack of evidence of discriminant validity of the two-factor reduced model. And also as a plausible option in theoretical terms as also suggested by other authors who have proposed a burnout second-order factor using MBI, CBI and OLBI (Marôco et al., 2008, 2014; Marôco and Campos, 2012). The bi-factor model presents equivalent fit, and some authors also proposed it as an alternative structure using MBI and the Job Burnout Scale (Wang and Gao, 2010; Mészáros et al., 2014; Morgan et al., 2014) although with problems in some cases (i.e., non-convergence, unsatisfactory unique proportion of variance explained of the observed scores). The second-order factor had very high structural weights, while the bi-factor model had only one factor loading above 0.50 on each for the two *specific* factors, pointing for clear insufficient proportion of the variance explained on the *specific* factors. Altogether, the obtained results seem to present evidence that favors the second-order model.

**Table 5:** OLBI models' goodness-of-fit indices.

**Model**	****χ**^**2**^**	***df***	***CFI***	***CFI*_***scaled***_**	***NFI***	***TLI***	***SRMR***	***RMSEA***	***RMSEA 90% CI***
Two-factor*^*C*^*	720.764	103	0.980	0.918	0.977	0.977	0.072	0.101	]0.094; 0.108[
Two-factor*^*MI, C*^*	514.098	85	0.986	0.937	0.984	0.983	0.064	0.093	]0.085; 0.101[
Unidimensional*^*MI, C*^*	748.051	86	0.979	0.913	0.976	0.974	0.079	0.115	]0.107; 0.122[
Bi-factor*^*C*^*	392.202	75	0.990	0.937	0.987	0.986	0.056	0.085	]0.077; 0.093[
Second-order*^*MI, C*^*	514.098	85	0.986	0.937	0.984	0.983	0.064	0.093	]0.085; 0.101[
Second-order*^*MI, C*^*	591.172	85	0.985	0.934	0.983	0.982	0.068	0.101	]0.093; 0.109[

MI *Modification indices applied (four residuals' correlations)*;

C *Calibration sample*;

V *Validation sample*.

This structure also showed cross-validity evidence, since it presented a good fit to the data also when using the validation sample ($\chi_{\left(85\right)}^{2}$ = 591.172; *p* < 0.001; *n* = 586; *CFI* = 0.985; *CFI*_*scaled*_ = 0.934; *NFI* = 0.983; *TLI* = 0.982; *SRMR* = 0.068; *RMSEA* = 0.101; *P*(rmsea ≤ 0.05) < 0.001; 90% CI ]0.093; 0.109[). The structural weights were high (γ = 0.93; *p* < 0.001).

#### Reliability of the Scores: Internal Consistency Evidence

To estimate the reliability of the scores on the various models, the complete sample was used. The internal consistency values of the two-factor first-order model reduced were high for the three coefficients (Table 6). They suggest very good validity evidence in terms of the reliability of the scores.

**Table 6:** Internal consistency of OLBI dimensions (two-factor reduced version).

**OLBI dimension**	**α_*ordinal*__*total sample*_**	**ω_*ordinal*__*total sample*_**	***CR*_*total sample*_**
Disengagement	0.91	0.87	0.90
Exhaustion	0.87	0.87	0.88
Total	0.93	0.92	–

The same was observed on the unidimensional model internal consistency estimates (*CR*_*burnout*_ = 0.93; α_*ordinal*_ = 0.93; ω_*ordinal*_ = 0.91). Based on a bi-factor model, the hierarchical omega was high (ω_H_ = 0.85) and omega hierarchical subscale (ω_*HS*_; Reise, 2012; Reise et al., 2013; Rodriguez et al., 2016b) were low (ω_*HS disengagement*_ = 0.08; ω_*HS exhaustion*_ = 0.20). The *specific* factors reliability score after controlling for the variance due to the general factor was clearly unsatisfactory, reinforcing the evidence in favor of the second-order model. The ω_*H*_ value was high (Rodriguez et al., 2016a), suggesting a strong influence of the latent variable common to the two factors.

The proportion of observed variance explained by the second-order factor after controlling for the uniqueness of the first-order factor (ω_*partial L*1_) was 0.93. The proportion of the variance of the first-order factors explained by the second-order factor (ω_*L*2_) was 0.91, and the proportion of the second-order factor explaining the total score (ω_*L*1_) was 0.86. Thus, the internal consistency of the second-order construct was indicative of very good values.

#### Measurement Invariance

To verify if measurement invariance holds, the complete sample was used. The fit to the data of each individual group was globally acceptable. The Brazilian sample had an acceptable fit ($\chi_{\left(85\right)}^{2}$ = 687.077; *p* < 0.001; *n* = 604; *CFI* = 0.982; *CFI*_*scaled*_ = 0.922; *NFI* = 0.980; *TLI* = 0.978; *SRMR* = 0.073; *RMSEA* = 0.109; *P*(rmsea ≤ 0.05) < 0.001; 90% CI ]0.102; 0.117[) as so did the Portuguese sample ($\chi_{\left(85\right)}^{2}$ = 494.338; *p* < 0.001; *n* = 568; *CFI* = 0.988; *CFI*_*scaled*_ = 0.943; *NFI* = 0.985; *TLI* = 0.985; *SRMR* = 0.065; *RMSEA* = 0.092; *P*(rmsea ≤ 0.05) < 0.001; 90% CI ]0.084; 0.100[). The fit of the Females sample was slight better ($\chi_{\left(85\right)}^{2}$ = 537.474; *p* < 0.001; *n* = 678; *CFI* = 0.989; *CFI*_*scaled*_ = 0.944; *NFI* = 0.987; *TLI* = 0.986; *SRMR* = 0.059; *RMSEA* = 0.089; *P*(rmsea ≤ 0.05) < 0.001; 90% CI ]0.082; 0.096[) whereas the Males sample fit was marginally worse ($\chi_{\left(85\right)}^{2}$ = 492.213; *p* < 0.001; *n* = 364; *CFI* = 0.979; *CFI*_*scaled*_ = 0.922; *NFI* = 0.975; *TLI* = 0.974; *SRMR* = 0.081; *RMSEA* = 0.115; *P*(rmsea ≤ 0.05) < 0.001; 90% CI ]0.105; 0.125[).

To assess if the same second-order latent model holds in each sex and country, seven nested models with indications of equivalence were used (Marôco, 2014). Full uniqueness measurement invariance was supported for countries (Table 7) based on the Cheung and Rensvold (2002) criterion (absolute Δ*CFI*_*scaled*_ ≤ 0.010) and on the Chen (2007) criterion (absolute Δ*RMSEA*_*scaled*_ ≤ 0.015). The Δχ^2^ criterion (Satorra and Bentler, 2001) demonstrated the second-order metric invariance. Since the Δχ^2^ criterion is too restrictive, we opted for the ΔCFI_*scaled*_ criterion. Results supported the structural invariance of the OLBI between Portugal and Brazil. The measurement invariance for OLBI among sexes (Table 7) was obtained, since full uniqueness measurement invariance was observed with the support of the Cheung and Rensvold (2002) criterion (absolute Δ*CFI*_*scaled*_ ≤ 0.010) and of the Chen (2007) criterion (absolute Δ*RMSEA*_*scaled*_ ≤ 0.015).

**Table 7:** OLBI second-order latent model measurement invariance.

**Model**	**χ^2^**	***df***	***CFI_***scaled***_***	***RMSEA_***scaled***_***	**Δχ^2^***_***scaled***_*	**Δ*CFI_***scaled***_***	**Δ*RMSEA_***scaled***_***
**COUNTRIES**							
Configural (factor structure)	1,192.415	167	0.932	0.129	–	–	–
First-order loadings invariance	1,243.826	180	0.931	0.125	57.168***	0.001	0.004
Second-order loadings invariance	1,246.442	183	0.935	0.120	0.696*^*ns*^*	0.004	0.005
Thresholds of measured variables	1,365.285	224	0.932	0.111	139.591***	0.003	0.009
Intercepts of first-order factors invariance	1,402.498	225	0.931	0.112	12.342***	0.001	0.001
Disturbances of first-order factors invariance	1,431.365	227	0.933	0.110	6.352*	0.002	0.002
Residual variances of observed variables invariance	1,683.368	242	0.934	0.105	73.549***	0.001	0.005
**SEX**							
Configural (factor structure)	1,029.687	165	0.940	0.122	–	–	–
First-order loadings invariance	1,069.647	180	0.939	0.118	46.568***	0.001	0.004
Second-order loadings invariance	1,069.647	181	0.939	0.118	< 0.001*^*ns*^*	0.000	0.000
Thresholds of measured variables	1,140.656	224	0.943	0.103	61.446*	0.004	0.015
Intercepts of first-order factors invariance	1,196.237	225	0.941	0.104	14.210***	0.002	0.001
Disturbances of first-order factors invariance	1,205.425	227	0.942	0.103	2.558*^*ns*^*	0.001	0.001
Residual variances of observed variables invariance	1,337.136	242	0.947	0.095	36.259**	0.005	0.008

ns *p > 0.05*;

* *p < 0.05*;

** *p < 0.01*;

*** *p < 0.001*.

### Sex's and Country's Burnout Latent Means Comparisons and Dimensions' Quartiles

Following the existence of full uniqueness measurement invariance, latent means can be compared. The results of the chi-square difference test suggest that weren't significant differences in burnout (Δχ^2^_*scaled*_ (1) = 1.110; *p* = 0.292; *d* = 0.067) among countries. There weren't also statistically significant differences among countries in relation to the burnout dimension (Δχ^2^_*scaled*_ (1) = 1.066; *p* = 0.302; *d* = 0.066). The quartiles, means, and SDs (raw values) for each sex within each country are presented in Table 8, these values are presented with the intent of providing population norms values.

Table 8Quartiles, means, and SDs for OLBI's dimensions (raw values) for countries and sexes.

**Table:** 

**OLBI dimension**	**Country**									
	**Brazil**					**Portugal**				
	**Multi-occupational (*****n*** **=** **604)**					**Multi-occupational (*****n*** **=** **568)**				
	***M***	***SD***	**25**	**50**	**75**	***M***	***SD***	**25**	**50**	**75**
Disengagement	2.46	0.89	1.71	2.43	3.14	2.56	0.87	1.86	2.43	3.14
Exhaustion	2.83	0.84	2.25	2.88	3.50	2.80	0.75	2.25	2.75	3.25
Burnout	2.66	0.80	2.07	2.67	3.20	2.69	0.73	2.20	2.67	3.20

**Table:** 

	**Sex**																			
	**Brazil (*****n*** **=** **528)**										**Portugal (*****n*** **=** **514)**									
	**Female (*****n*** **=** **355)**					**Male (*****n*** **=** **173)**					**Female (*****n*** **=** **323)**					**Male (*****n*** **=** **191)**				
	***M***	***SD***	**25**	**50**	**75**	***M***	***SD***	**25**	**50**	**75**	***M***	***SD***	**25**	**50**	**75**	***M***	***SD***	**25**	**50**	**75**
Disengagement	2.50	0.90	1.79	2.43	3.14	2.34	0.86	1.71	2.29	2.86	2.52	0.89	1.86	2.43	3.14	2.63	0.88	2.00	2.57	3.29
Exhaustion	2.88	0.85	2.25	3.00	3.50	2.66	0.80	2.13	2.63	3.25	2.87	0.76	2.38	2.88	3.38	2.64	0.72	2.19	2.75	3.13
Burnout	2.70	0.82	2.07	2.73	3.27	2.51	0.77	1.93	2.53	3.00	2.71	0.75	2.20	2.73	3.20	2.63	0.73	2.13	2.67	3.17

### Validity Evidence Based on the Relations to Other Variables

Burnout can be conceptualized as being the opposite of work engagement (Halbesleben and Demerouti, 2005). The UWES-9 second-order latent factor model presented a good fit ($\chi_{\left(24\right)}^{2}$ = 175.820; *p* < 0.001; *n* = 1,104; *CFI* = 0.999; *CFI*_*scaled*_ = 0.992; *NFI* = 0.999; *TLI* = 0.999; *SRMR* = 0.028; *RMSEA* = 0.076; *P*(rmsea ≤ 0.05) < 0.001; 90% CI ]0.065; 0.086[). All factor loadings were statistically significant as also one added residuals' correlation from item 1 and 2 (*r* = 0.76). The internal consistency reliability estimates were good both for the first-order model (ω_*vigor*_ = 0.84; ω_*dedication*_ = 0.92; ω_*absorption*_ = 0.88; ω_*total*_ = 0.96) and for the second-order (ω_*partial L*1_ = 0.96; ω_*L*1_ = 0.94; ω_*L*2_ = 0.97).

The first-order models of each instrument were used to establish correlations among the first-order latent variables (Table 9), and the second-order models of the instruments were used to analyze the correlation among the respective second-order latent variables (*r*_burnout*workengagement_ = −0.85). The obtained correlation between the latent OLBI and UWES dimensions was negative and moderate to high, demonstrating the divergent validity of the measures obtained with OLBI (*burnout*) and the UWES-9 (*work engagement*).

**Table 9:** Correlations between OLBI's and UWES-9's latent variables.

	**Vigor**	**Dedication**	**Absorption**	**Disengagement**	**Exhaustion**
Vigor	1	–	–	–	–
Dedication	0.98	1	–	–	–
Absorption	0.88	0.92	1	–	–
Disengagement	−0.85	−0.87	−0.76	1	–
Exhaustion	−0.73	−0.63	−0.56	0.83	1

*All correlations were statistically significant p < 0.001; df = 1,102*.

## Discussion

Regarding the psychometric properties of the OLBI, results of this study provide evidence of the two-factor structure of the original instrument, having convergent validity evidence and good goodness-of-fit indices except for RMSEA value, which can be indicative of moderate errors of approximation in the population. Nevertheless, this value had a narrow confidence interval, that reflects a good precision of the model fit in the population (MacCallum et al., 1996). However, the discriminant validity evidence was not satisfactory (H1), which led us to test a possible second-order latent factor. Item 13 was removed from the tested version. Problems with item 13 also have been reported by Chevrier (2009). Item 13 has also been deleted from the OLBI-S proposal for Portuguese and Brazilian students (Campos et al., 2012) and OLBI's Malay version for students (Mahadi et al., 2018). In the Brazilian version for workers, that item was removed from the proposed reduced 13-item version (Schuster and Dias, 2018). Its removal was also suggested from the Italian version (Estévez-Mujica and Quintane, 2018). Other authors found that item 13's removal increased the disengagement internal consistency (Baka and Basinska, 2016). In the OLBI reduced Russian version (Smirnova, 2017), that item was the only one that lacked statistically significant loading, also being removed. In the Swedish version, it was the item with the lowest loading (λ_*item* 13_ = 0.38) (Peterson et al., 2011). Item 13's content, “*This is the only type of work that I can imagine myself doing*,” did not seem to make sense within today's economic and professional context, where careers are so uncertain and the number of different employers across a career is increasing (Savickas, 2012). Furthermore, the sample is composed mainly of younger workers, most of whom have a higher education. This can contribute to a perception of more control over their career and desire for more professional experiences rather than maintaining the same employer for a long period. Additionally, four correlations within pairs of residuals' belonging to the same factor were added. Such modifications mean that some unwanted or unexplained source of variance exist outside of the original model, which can be due to various reasons (e.g., lack of comprehension of the items or an unwanted theoretical trait present in that factor) and as such is speculative (Cote et al., 2001). Nevertheless, it seems plausible that theory can be slightly imprecise, and since the model (second-order) passed through the cross-validation approach and reinforced the validity evidence obtained with the calibration subsample. The correlation between the two OLBI's dimensions was high; this finding is shared by other studies which found correlation values between OLBI's factors similar to ours (Khan et al., 2016). Those findings also pointed to unsatisfactory discriminant validity evidence between disengagement and exhaustion.

Our internal consistency estimates presented very good values supporting H2, in line with most of the studies which evaluated this kind of estimate (Table 1). Still, this study goes one step further, since it obtained estimates that gave evidence about the reliability of the second-order latent factor and the reliability of a potential bi-factor model (ω_*H*_).

We admitted a possible second-order factor which was confirmed (H3) since the goodness-of-fit indices were not worse than the reduced version of the first-order model. The second-order model was also compared with a bi-factor model, which also didn't present *meaningful* better fit (i.e., Δ*CFI*_*scaled*_ < 0.01) to the data than the second-order model. The bi-factor model has various advantages (Reise et al., 2010), although—in this study- those potential advantages weren't confirmed by the obtained results. Since the unique variance explained by the *specific* factors of (after controlling for the variance due to the general factor) wasn't satisfactory (Rodriguez et al., 2016b). Additionally, empirical observations strongly suggest the second-order model presented high values of structural weights both on the calibration as on the validation subsamples. Regarding the existent theory, some authors proposed burnout as a second-order factor (Taris et al., 1999; Shirom and Melamed, 2006; Marôco et al., 2008) the same applies to the bi-factor model with some authors preferring it in relation to hierarchical model (Mészáros et al., 2014). However, if there wasn't prior knowledge from the field regarding burnout conceptualization, exploratory bi-factor analysis (Jennrich and Bentler, 2011) or bi-factor exploratory structural equation modeling (Morin et al., 2015) cloud be also suitable analyses. If one desires to analyze bi-factor models outside the standard CFA procedures (e.g., setting to zero paths from latent constructs to indicators that are not theoretically associated) can adopt the already referred bi-factor exploratory structural equation modeling or the bi-factor Bayesian structural equation modeling (Golay et al., 2013) analyses. Marôco and Campos (2012) have proposed the a second-order interpretation of burnout, although using a different instrument, the MBI. In other study, Marôco and Campos (2012) made the same suggestion using the MBI, CBI and OLBI. This suggests that, besides the two OLBI first-order factors, there is a higher-order more general burnout dimension. The obtained findings contribute to the study of OLBI and the burnout dimensionality, maintaining the two-order structure, but also suggesting a second-order latent, which brings a novelty to this study, since this was proposed for the first time for OLBI using a sample of workers.

The OLBI presented measurement invariance for sex and country supporting H4. To the best of our knowledge, this was the first time measurement invariance was assessed across sexes for the full OLBI instrument, since Foster (2015) only tested measurement invariance among sex on the separate factors. No study has found testing measurement invariance among countries, bringing a novelty to this study.

No statistically significant differences were found between sexes for burnout (H5), which shows that each sex experiences that dimension in the workplace similarly. Females are known for having higher levels of negative emotional states than males (Kessler et al., 2005); in consequence, females usually score higher than males on exhaustion (Purvanova and Muros, 2010; Kumar and Mellsop, 2013; Pu et al., 2017; Schadenhofer et al., 2018). However, a meta-analysis (Purvanova and Muros, 2010) found that males usually have higher depersonalization levels than females. Altogether, the differences reported on previous studies seem to be annulling each other in terms of the general second-order factor. The relationship between burnout dimensions and sex is not always clear. O'Connor et al. (2018) suggested in a meta-analysis on burnout in health professionals, that the burnout dimensions and sex have an inconsistent relationship. A similar finding was reported by Estévez-Mujica and Quintane (2018), which found no relationship between exhaustion, disengagement, and burnout and sex in a sample of research and development workers. A recent study with Portuguese health professionals at the national level, found no significant differences among sexes in terms of burnout (Marôco et al., 2016). For the country factor, no significant differences were observed for the burnout latent variable (H5). These results are in line with the findings of a comparative study among Portuguese and Brazilian health professionals using the MBI (Dias et al., 2010), no significant differences were found between the burnout dimensions. Western European countries seem to present lower average burnout scores than in other parts of the world (Golembiewski et al., 1996). These differences can be due to cultural differences (Golembiewski et al., 1993; Maslach et al., 2001), In some countries, as Brazil, such differences can also occur between regions of the same country as a result of being such a big and culturally-mixed country (Hofstede et al., 2010b). Differences between Portugal and Brazil seem to exist at the individualism level, with Portugal being more collectivist and with smaller power distance (Hofstede et al., 2010a). In Brazil, employers seem to be more risk-taking than their Portuguese counterparts (Silva et al., 2009). In other words, Portugal has larger avoidance to uncertainty values (Hofstede et al., 2010a). In Brazil, organizations operate through general rule as much as through personal relationships (Garibaldi de Hilal, 2009); whereas in Portugal, the work relations appear to be more impersonal and formal (Dias et al., 2010). Brazil presents larger indulgence values than Portugal (Hofstede et al., 2010a); the Brazilian culture also reflects ambiguity and double-edged ethics (Garibaldi de Hilal, 2006). Brazilian organizations seem to perceive responsibility toward employees as one of the less important business priorities (Hofstede et al., 2010a), which can lead to poorer attention to work conditions.

Regarding the last hypothesis, the validity evidence based on the relations with other variables was good (H6), since the presented correlations were moderate to high between OLBI and UWES factors. Other studies found Pearson's correlation value −0.55 between the work engagement (UWES) score and OLBI's disengagement factor, and −0.48 between work engagement (UWES) and OLBI's exhaustion (Bosman et al., 2005). In the present study, a higher work engagement correlation with disengagement than with exhaustion was also found. The produced results were in line with the findings of Petrović et al. (2017), who found similar correlation values between the instruments' dimensions and a higher correlation between the pairs of variables previously referred to González-Romá et al. (2006). In other words, the correlations between vigor and exhaustion were higher than with the other UWES variables, and the same for the disengagement-dedication pair. This study's results are in the same direction, reinforcing the higher association between disengagement and dedication than between disengagement and vigor or absorption, and a higher association between exhaustion and vigor than between exhaustion and dedication and absorption.

Our results confirmed the four of the five hypotheses and gave us, globally, good validity evidence regarding this OLBI version. However, since H1 had unsatisfactory validity evidence in terms of discriminant validity, this study brought a novelty (in terms of OLBI dimensionality studies); therefore, a second-order latent model was proposed, which is admissible from a theoretical and practical perspective. Thus, it is suggested that OLBI can be used to compare burnout levels among samples with different occupations and sexes from Portugal and Brazil. However, since the proposed version is a reduced version, it must be said that the reduced version must be tested in independent samples of the same populations (Marôco, 2014).

### Theoretical Implications

Burnout seems to be a second-order factor that loads on two first-order factors, disengagement, and exhaustion—which are burnout's core dimensions. This underlines the expansion of the burnout domain beyond the exhaustion affective component (Halbesleben et al., 2004). Female and male workers experience similar levels of burnout, the same happens among Portuguese and Brazilian workers. The obtained results demonstrate that the absence of differences in burnout between sexes and countries suggest that the work experiences in terms of stress are similar in both samples. The observed differences between sexes reported on other studies are small (Purvanova and Muros, 2010) and might be related to other factors—namely family and workload (Bekker et al., 2005; Langballe et al., 2011).

### Practical Implications

To have instruments with good validity evidence, it is mandatory to have confidence in the obtained measures. OLBI can help to establish comparisons between sexes, and countries. However, one should be aware of the different versions and of the quality of the evidence provided in each study. Also, OLBI can be useful and practical, since it is a freely available self-report psychometric instrument which can contribute to studies where the impact of companies' interventions are studied (Gíslason and Símonardóttir, 2018). In fact, there is evidence that occupational stress can be reduced with specific interventions (Ruotsalainen et al., 2015). The development of a family-friendly work environment should be approached, allowing one a focus on the importance of a balance between work and life (Lo, 2003; Rubino et al., 2013). Giving workers paid sick days, medical and family leave insurance programs, and greater control over their schedule (Appelbaum et al., 2014) are good suggestions to improve the balance between work and home activities. However, any change in organizational practices without a corresponding change in social attitudes will not be enough (Field and Bramwell, 1998). Prevention strategies should consider the social and individual level of those that will receive them (Maslach and Leiter, 2017).

### Limitations

This study used two convenience samples. It had no other psychological measures besides burnout and work engagement, which would allow better assessment of the validity evidence based on the relationship between other variables—namely, predictive, concurrent, and discriminant evidence (American Educational Research Association, 2014). For example, a concurrent burnout measure which would allow verification of the concurrent validity evidence between different burnout instruments, as some studies have done (Demerouti et al., 2003; Marôco and Campos, 2012) would have been useful. Another limitation is that culture-specific aspects of stress were not assessed, which could explain some of the observed differences.

### Future Research

Further studies using OLBI should test its concurrent validity evidence with other burnout instruments (American Educational Research Association, 2014), something that has been tested with success in the Portugal-Brazil version for students (Campos et al., 2012) and in the Greek version for workers (Demerouti et al., 2003). The Portugal-Brazil OLBI version should be tested in samples of specific occupational groups (e.g., Armed Forces Occupations, Craft and Related Trades Workers, Skilled Agricultural, Forestry and Fishery Workers, Plant and Machine Operators and Assemblers, and Elementary Occupations), of whom there was not a satisfactory number in the sample collected for this study. The same applies to lower academic levels, which showed unsatisfactory frequency in this study. Future research should also assess the family load together with the workload to understand the family-work interaction regarding burnout.

## Conclusion

Initially, burnout research was linked to human services occupations: thus, sex was not a concern since most employees in this area were female. This study compared burnout levels between sexes in two different countries, and simultaneously adapting a specific instrument that allows establishing direct comparisons between countries.

OLBI offers various advantages over other instruments that can measure burnout, and the obtained findings focus on the utility of this inventory to compare burnout among sexes and countries using samples from Portugal and Brazil. The instrument showed validity evidence based on the internal structure and on the relation with other variables (work engagement and its first-order dimensions). Altogether, the proposed OLBI version appears to be a valid alternative to assess burnout and establish rigorous comparisons between Portuguese and Brazilian workers. The differences between sexes seem to non-existent. Burnout differences reported in other studies seem to be related to other factors, such as work and family load, as previous research suggested (Bekker et al., 2005; Langballe et al., 2011).

## Author Contributions

All authors of this research paper have directly participated in the planning, execution, or analysis of this study. More specifically, JS wrote the first draft, and with JM performed all statistical analysis and its discussion. JS and SP discussed cross-cultural topics, and JS and CQ discussed theoretical framework.

### Conflict of Interest Statement

The authors declare that the research was conducted in the absence of any commercial or financial relationships that could be construed as a potential conflict of interest.

## Footnotes

^1^Bi-factor or bifactor model are both acceptable forms, we adopted the first one, originally used by Holzinger and Swineford (1937).
